# Genetic Epidemiology and Preventive Healthcare in Multiethnic Societies: The Hemoglobinopathies

**DOI:** 10.3390/ijerph110606136

**Published:** 2014-06-11

**Authors:** Piero C. Giordano, Cornelis L. Harteveld, Egbert Bakker

**Affiliations:** Human and Clinical Genetics Department, Leiden University Medical Center, P.O. Box 9600, Leiden 2333 ZC, The Netherlands; E-Mails: c.l.harteveld@lumc.nl (C.L.H.); e.bakker@lumc.nl (E.B.)

**Keywords:** hemoglobinopathy, thalassemia, sickle cell disease, prevention, ethnicity

## Abstract

Healthy carriers of severe Hemoglobinopathies are usually asymptomatic and only efficiently detected through screening campaigns. Based upon epidemiological data, screenings have been offered for decades to populations of endemic Southern Europe for primary prevention of Thalassemia Major, while for many populations of the highly endemic African and Asian countries prevention for Sickle Cell Disease and Thalassemia Major is mainly unavailable. The massive migrations of the last decades have brought many healthy carriers of these diseases to live and reproduce in non-endemic immigration areas changing the epidemiological pattern of the local recessive diseases and bringing an urgent need for treatment and primary prevention in welfare countries. Nonetheless, no screening for an informed reproductive choice is actively offered by the healthcare systems of most of these welfare countries. As a consequence more children affected with severe Hemoglobinopathies are born today in the immigration countries of Northern Europe than in the endemic Southern European area. Following the Mediterranean example, some countries like the UK and The Netherlands have been offering early pregnancy carrier screening at different levels and/or in specific areas but more accessible measures need to be taken at the national level in all immigration countries. Identification of carriers using simple and inexpensive methods should be included in the Rhesus and infectious diseases screening which is offered early in pregnancy in most developed countries. This would allow identification of couples at risk in time for an informed choice and for prenatal diagnosis if required before the first affected child is born.

## 1. Introduction

*Genetic epidemiology and public health*: There are quite a few examples of genetic diseases that are more prevalent in one population then in another. Just to mention the common one: Cystic Fibrosis (CF), which is most prevalent in people of European heritage; Tay-Sachs disease (TSD) is frequent among Ashkenazi Jews; Hereditary Hemochromatosis (HFE), most common among people of Northern European Celtic descent and the most common of them all and subject of this review, the Hemoglobinopathies (HBP) better known in the two major forms as Sickle-Cell Disease (SCD) and Thalassemia Major (TM). 

Not just by chance these genetic diseases are autosomal recessive, meaning that the conditions are not sex linked and that carriers are just healthy persons, with or without some minor symptoms. Recessive means also that when two carriers get children, the traits will be inherited according to the Mendelian law of inheritance and that at each and every pregnancy the statistical outcome will be 25% chance to have a severely affected child, 50% chance to have a healthy carrier just like the parents and 25% chance to have a child not carrier. In origin HBPs were restricted to populations living in the tropical and subtropical regions of the “Old World” infested for thousands of years by malaria and by *malaria tropica* in particular. This brings us to the question why these diseases are prevalent in specific populations. 

*Diseases, selection mechanisms, epidemiology and prevention*: The key mechanism at the base of evolution is the selection of random gametic mutations passed to the progeny to be tested for their beneficial or adverse effect. If a new recessive mutation gives an advantage to the offspring (better genetic fitness to survive and reproduce) in a particular environment then it will be passed more frequently to the following generations and become prevalent in that particular population living in that particular environment. In spite of the adverse effects, recessive trait advantageous in the carriers will then become more and more common until equilibrium is reached between advantages and disadvantages at a stabilized level that will characterize the epidemiology of the severe disease in a particular population. 

Due to the severity of recessive diseases that manifest in the children of healthy carrier couples, prevention and carrier detection are important public health issues. Morbidity prevention can be offered by newborn screening to reduce the severe symptoms in the born child and primary prevention can be made available to couples at risk to prevent the birth of a severely affected child. Primary prevention can be prospective if offered before the first affected child is born or retrospective if made available after the birth of the first affected offspring. 

*Cystic Fibrosis*: Several selection hypotheses have been proposed to explain the epidemiology of CF. Being a disease that disrupts the water balance in the body, the most acceptable explanation seems to be a better chance of survival during epidemics of infectious diseases causing life threatening diarrhea such as cholera and typhoid, which are known to have infested Europe for centuries [1]. Whether this is the only cause of CF epidemiology in Northern Europeans is not sure, but the fact remains that to date, about one in 25 persons of European descent is a carrier and that the incidence of the severe disease in these populations can reach one in 2,500 to 3,000 newborn. 

But for some milder forms, the symptoms of CF usually manifest within the first year of life, causes severe impairment of the respiratory and digestive system and the available cures may prolong the life of the patient up to early adult age. Due to the severity of the disease, primary prevention for CF is offered in many countries but mainly retrospectively, to couples at risk after the birth of the first affected child. But for a limited “population screening” among Ashkenazi-Jewish [2] no national screenings campaign for prospective prevention have been implemented on a large scale thus far because of the expensive tests needed to identify symptomless carriers at the DNA level. New developments in molecular diagnostics will hopefully offer a financeable method for screening either before marriage/conception or early in pregnancy in the near future. 

*Hemochromatosis*: The prevalence of Hemochromatosis (HFE) among people of Northern European Celtic descent suggests that the disease may also have given some advantage to the carriers. A sensible theory to explain the epidemiology of the disease seems that a higher dietary iron reserve might have given a reproductive advantage to carriers in particular during pregnancies in harsh famine winters. Among northern and central Europeans over 12% of the indigenous people are carriers [3]. Because of late onset, screening for primary prevention seems only appropriate for those rare and severe cases when iron overload begins before birth [4]. 

*Tay-Sachs Disease*: Not all recessive traits common in a specific population can be explain with a genetic advantage and a positive selection mechanism. Due to founder effect, genetic drift and inbreeding 57% of the population of the island of Tristan da Cunha has been reported with different degrees of asthma [5] and high frequency of retinitis pigmentosa [6]. This brings us to Ashkenazi Jews, particularly affected with Tay-Sachs disease. No advantages have been found for TSD carriers and some authors suggests indeed a combination of founder effect, genetic drift, and differential migration patterns [7]. To these three factors one may easily add the effect of cultural isolates and endogamy. 

Kabak *et al*. report that screening for TSD has been well organized among Ashkenazi-Jewish for many years, decreasing the incidence of the disease by more than 90% [8] and that practical, social, and ethical complexities had to be tackled and that educational and counseling components had to be properly explained before and after screening in total confidentiality. The same author wondered about the fact that wide-scale testing and screening could be felt, in some systems, as a conflict of interest on the part of entrepreneurial scientists, clinicians, and institutions. 

Mitchel *et al*. presented in 1996 the results of a 20 years program for education, screening, and counseling of senior-high-school students in populations at high risk for Tay-Sachs and beta-thalassemia diseases in Montreal [9]. Screening 14,844 Ashkenazi-Jewish students they identified 521 HexA-deficient carriers (frequency 1:28). The corresponding data for the concomitant beta-thalassemia program were 25,274 students (mainly of Mediterranean origin) representing 67% of the cohort with 61% voluntary participation in the screening phase (693 carriers; frequency 1:36). Virtually all the carriers identified in the high-school screening program remembered their status, had their partner tested and took up the options for reproductive counseling/prenatal diagnosis. Incidence of the two diseases has fallen by 90%–95% over 20 years in Canada. 

## 2. Hemoglobinopathies

While TSD carriers can be identified retrospectively after the birth of the first affected child or prospectively before marriage or early in pregnancy at the enzymatic and/or DNA level, HBP carriers can be identified at any time with a simple, fast, and inexpensive Hb separation and measurement on high performance liquid chromatography (HPLC) or capillary electrophoresis (CE), the modern version of the classic Hb electrophoresis [10]. With an incidence of over 350,000 affected newborn p.y. HBP are the most common autosomal recessive disease worldwide [11]. The conditions are caused by mutations that impair or modify the expression of the globin genes causing respectively lack of globin synthesis (thalassemias) or structural defects (abnormal hemoglobins). But for some semi-dominant mutation, carriers are either slightly anemic (thalassemia) or asymptomatic (most abnormal hemoglobins) and thus healthy. Conversely, children of healthy carrier couples have 25% chance of being affected with the severe forms Thalassemia Major (TM) and/or Sickle Cell Disease (SCD). While thalassemias can be caused by more than 450 mutations, almost 1200 mutations have been reported changing the structure of the alpha-like and beta-like globin gene products that are needed to build up the hemoglobin tetramers, the essential oxygen transport proteins during embryonic, fetal and postnatal life. 

Among this broad spectrum of mutations some are very frequent and population specific. As mentioned above, specificity is explained by the random occurrence of a particular mutation and frequency by the advantage given against malaria in tropical or subtropical region of the old world by that particular mutation. Random HBP mutations might very well be archaic but positive selection have probably started with the beginning of agriculture around 10,000 years ago when man had to share his surrounding with the anopheles mosquitos. In these surroundings children carriers of the trait had better chances to survive malaria during infancy, to reproduce and to pass the trait to the next generation increasing the gene frequency. This mechanism has generated carrier frequencies of structural hemoglobin variants like HbS (the main cause of SCD) of around 40% in some regions of central Africa. Equally the HbE variant, alpha thalassemia and beta thalassemia have reached respective frequencies of 40%, 50%, 8% in some highly endemic regions of Thailand, the Arabian Peninsula and the Mediterranean. 

Due to the high incidence and the severity of the disease, pioneering Mediterranean countries have provided carrier screening and primary prevention mainly for TM, the most frequent form in the area. In countries like Italy [12,13], Greece [14], France [15], Cyprus [16] Israel, [17,18], Turkey [19] and Egypt [20], the incidence of the disease have been reduced substantially and in some areas by more than 90%. The tools used are information, premarital/preconception screening or screening in early pregnancy identifying couples at risk in time for an informed reproductive choice. Ongoing screening programs are summarized in Table 1, adapted and updated from Cousens *et al*. [21].

**Figure 1:**
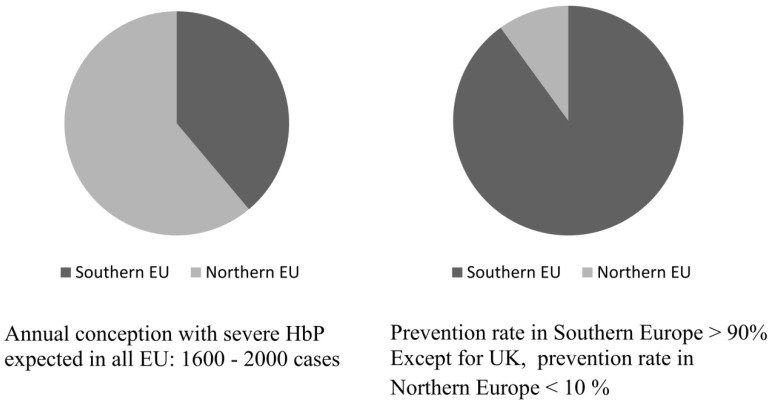
Incidence and prevention in endemic southern and in non-endemic northern Europe.

Today, the historical epidemiology of these diseases has changed dramatically and healthy carriers are present all over the world, while more affected children are born in the non-endemic immigration areas of Northern Europe than in the endemic Mediterranean countries. The growing incidence in non-endemic areas is not only caused by the increasing number of immigrant carriers and endogamous marriages but by the fact that primary prevention, often available in the countries of origin, has not been properly introduced in the healthcare systems of most non-endemic immigration countries. Figure 1 adapted from Modell *et al*. [22]. 

**Table 1:** Global preventive carrier screening for Hemoglobinopathy before conception or early in pregnancy and newborn screening reporting carriers, today. Countries with longest experienced at the top. NBS= Newborn screening; * School and pregnancy screening interrupted in 1986 and 1995 respectively. # Mandatory before Church marriage only. & Mandatory before marriage. @ At secondary or high school level.

Country (Region) Screening type	Extent: Universal (U) Regional (R)	Pre-conception: Mandatory (M) Voluntary (V)	Early in pregnancy Mandatory (M) Voluntary (V)	NBS: Carriers reported: Y, N
Cyprus	U	M for Turkish &	V Turkish	---
		M for Greeks #	V Greeks	
Greece	U	V	V	---
Italy (Sardinia)	U	V	V	---
Italy (Latium)	U	V (School) @	V	---
Italy (North East)	R	V	V	R, Y
France (Southern)	R 1978–85	V (School) * @	V*	U, Y
Canada	R 1979–92	V (School) @	V	R, Y
Israel	U	V	V	---
Maldives	U	V	V	---
Taiwan	U	V	V	---
China	R	V	V	---
Iran	U	M &	V	---
India	R	V	V	R, Y
Palestine	U	V	V	---
Turkey	U	V	V	---
Saudi Arabia	U	M &	V	---
Egypt	---	If requested	V	---
UK	U	If requested	V	U, Y
The Netherlands	R	If requested	V	U, Y
Germany	---	If requested	If requested	---
Austria	---	If requested	If requested	---
Swiss	---	If requested	If requested	---
Belgium	---	If requested	If requested	R, Y
Denmark	---	If requested	If requested	---
Sweden	---	If requested	If requested	---
Norway	---	If requested	If requested	---
Spain	---	If requested	If requested	R, Y
Portugal	---	If requested	If requested	---
USA	---	If requested	If requested	U, Y
Latin America	---	If requested	If requested	R, Y
Australia	---	If requested	If requested	---
Japan	---	If requested	If requested	---
Africa	---	Not available	Not Available	Some pilots

*Newborn screening*: During the last decade HBP’s have been included in the ongoing newborn screening (NBS) in a number of Northern European countries, in the USA and as pilot experiments in some South American and African states [23,24,25,26,27] (Table 1). NBS has been originally planned following the model used for metabolic diseases like Phenylketonuria (PKU), and thus for morbidity (secondary) prevention providing early treatment directly after birth, before irreversible organ damage occurs. 

Strictly speaking SCD and TM do not need to be diagnosed at birth for urgent treatment directly after birth. The affected newborns are perfectly healthy at birth and stay symptomless for several months, until the fetal hemoglobin has disappeared. The pathological symptoms begins when postnatal HbA cannot be formed due to the total absence of normal beta globin chains (TM) or when red cells begin to sickle due to the increasing presence of HbS due to the following common genotype combinations (Hb S/S, HbS/C, S/D, S/E, S/beta thal, *etc.*…). 

Nevertheless, thanks to early diagnosis, NBS allows morbidity (secondary) prevention to be started in time about 6 months after birth. During the first 6 months one may confirm the disease at the genotype level, make a genotype/phenotype correlation and a prognosis and may tailor the best supportive treatment to be offered when the symptoms begin. In the same period parents can be counseled in a genetic center and become able to make a retrospective reproductive choice for the next child. Also parents at risk who had a carrier can be counseled for prospective primary prevention in the future. Obviously couples at risk who had a “non-carrier” (25%) are not identified by NBS until the first affected child is born. The benefit of NBS consists mainly of a lower risk of mortality during the first few years of life when patients may die of an acute condition while not yet diagnosed. This benefit comes with increased treatment costs which are already considerable for these patients and can be estimated around 2 million US $ per average patient treated in developed countries [28].

While one could have expected that some primary retrospective or prospective primary prevention could have been reached after NBS, data have shown that this is not the case, at least not in The Netherlands, where after 7 years of NBS screening, no reduction of incidence has been monitored. The reasons can be searched in the partial report of carriers (HbS only), in the possibly directive counseling if focused on treatment rather than on explaining the severity of the progressive diseases, in the lack of motivation of part of the badly informed target population [29] and last but not least in potential conflict of interests that, as suggested by Kabbak, could be felt in some systems, as conflicts of interest on the part of entrepreneurial scientists *vs.* clinicians, and institutions [8]. Politics might be afraid of the public opinions of conservative voters if a screening campaign, which may imply medical abortion, is approved. Scientist might be tempted to propose treatment or prevention technologies more for their scientific reward than for the benefit of the patients and couples at risk. Prevention might reduce considerably the number of chronic patients in need of expensive treatment which might stimulate pediatricians to focus on treatment rather than on prevention. In addition, counseling given by pediatricians is bound to be optimistically directive not to distress the parents describing the severity of these progressive incurable diseases in the years to come. All these factors may prevent NBS from being a useful tool for at least partial retrospective and prospective primary prevention and therefore screening should be offered much earlier to couples at risk also in non-endemic immigration countries with large multiethnic populations. In fact, the epidemiology of HBP in most non-endemic immigration countries has already reached levels that, according to WHO recommendation, justify the implementation of carrier screening for prospective primary prevention [30]. 

## 3. Implementation of Carrier Screening

### 3.1. Screening at School Level

Beside the Montreal experience mentioned above [9], two successful programs have been implemented in endemic Southern Europe (Table 1). A screening in Marseille [31] has been shown successful, but the program has been stopped due to lack of funds and the very low incidence in the meanwhile well informed population. The Italian program in the Latium region is an exemplary one and is the longest ongoing [12] (Table 1). The reason for screening is explained to parents and students, herewith reaching two generations and providing cascade screening for more family members as well and is therefore welcome by the local population. In Latium the incidence among natives has been zero for almost a decade, while it has increased among those recent immigrants that have not been reached by the screening protocol as yet.

Although feasible, no school screening programs are available or have been planned in non-endemic immigration countries eventually coupled to a biology lesson. Ethnic discrimination could become a problem if the fact that everybody is carrier of recessive diseases regardless the ethnic origin is not well explained to the students. 

### 3.2. Pre-Conception Screening

Screening young adults before pregnancy or marriage allows more prevention options, from adapting partner choice to remaining childless, using gamete donation, pre-implantation diagnostics (PGD) or, still the most common, prenatal diagnosis (PD). 

As mentioned above, screening before marriage or conception has been offered to couples at risk in endemic countries and in different ways. The mandatory way has been ongoing for a long time in Christian Greek Orthodox Cyprus where in order to marry in the Church, couples need among the usual documents also a lab result for HBP (Table 1). The same mandatory rule is applied in Islamic countries like Iran and the UAE’s before legal marriage. While in Cyprus there is no interference on partner choice, in Iran adapting partner choice has been advised as a first option and PD with legal medical abortion as a second. In the UAE’s adapting partner choice is the only advice provided while PD and (illegal) medical abortion are possibly done abroad. 

But for some Jewish minorities who are used to screen before marriage or pregnancy, screening is not customary among immigrants in most non-endemic countries where in fact there are not many structures available for a pre-conception / pre-marital screening that could operate at the national level. Conversely, screening early in pregnancy is socially accepted and is the most rational alternative already routinely offered at the national level for other inborn diseases. 

### 3.3. Screening Early in Pregnancy

Screening early in pregnancy does not stigmatize the female partner (a risk in some cultures when screening before marriage) but on the contrary, it involves both parents in the process of choosing not to have a severely affected child. The disadvantage is however that screening early in pregnancy leaves as only prevention option PD and medical abortion. 

Deciding to interrupt a pregnancy, even if in an early fetal stage and knowing to bear a fetus that will become severely affected, is a difficult and emotional process for most couples involving their moral feelings and/or religious believes. Religious leaders have different views on this matter, some may reject medical abortion regardless the situation while others may consider it as an act of mercy [31]. 

While some parents may not feel restricted in exploring there medical options, others may feel religiously conflicted between their medical options and the decision to have an affected child who will likely have a shorter life with a severe progressive disease which can be treated but which is still incurable. 

Following the Mediterranean example, some immigration countries like the UK and the Netherlands are offering carrier screening early in pregnancy at different levels and/or in specific areas. Since in all non-endemic immigration countries all women are offered Rhesus and infectious diseases screening at the first pregnancy control, structures are available for offering HPB screening in early pregnancy that could operate at the national level (Table1). 

*Cost/benefit analysis*. Koren *et al*. have recently calculated that the expenses for running a thalassemia prevention program in Northern Israel during one year (2011) was about 400,000 US $, while the cost of basic treatment of a single patient for an average life expectancy was about 2,000,000 US $ [28]. Other authors have commented that primary prevention of HBP is not only financially justifiable but it is also an act of mercy sparing suffering to potential patients and families [32]. 

## 4. Conclusions

This paper is meant to make public health authorities aware of the fact that measures at the national level should be taken to offer an informed reproductive choice to multi-ethnic couples at risk in non-endemic immigration countries. Since all women are offered Rhesus and infectious diseases screening at the first pregnancy control in most countries, identification of HBP carriers using simple and inexpensive methods should be included in this screening to identify healthy couples at risk at the national level and in time for an informed choice and for prenatal diagnosis if required before the first affected child is born [33]. 
