# *Correction*: Giordano, P.C.; *et al.* Genetic Epidemiology and Preventive Healthcare in Multiethnic Societies: The Hemoglobinopathies. *Int. J. Environ. Res. Public Health* 2014, *11*, 6136–6146

**DOI:** 10.3390/ijerph111212367

**Published:** 2014-11-28

**Authors:** Piero C. Giordano, Cornelis L. Harteveld, Egbert Bakker

**Affiliations:** Human and Clinical Genetics Department, Leiden University Medical Center, P.O. Box 9600, Leiden 2333 ZC, The Netherlands; E-Mails: c.l.harteveld@lumc.nl (C.L.H.); e.bakker@lumc.nl (E.B.)

The authors wish to add the following amendment and correction on their paper published in IJERPH [1]: 

Page 6139, Line 3 in Paragraph 3: “…Israel, [17,18],…” should read “…Israel and Palestine [17,18]…”.

The authors would like to apologize for any inconvenience caused to the readers by these changes.
